# Sex-specific assumptions underlie cardiovascular digital twin technologies: A narrative review

**DOI:** 10.1371/journal.pdig.0001692

**Published:** 2026-08-31

**Authors:** Bettine G. van Willigen, Henk A. Marquering, Wouter Huberts, Joris R. de Groot

**Affiliations:** 1 Department of Clinical and Experimental Cardiology, Amsterdam University Medical Centre, University of Amsterdam, Heart Centre, Amsterdam Cardiovascular Sciences, Amsterdam, the Netherlands; 2 Department of Biomedical Engineering & Physics, Amsterdam UMC, Amsterdam, The Netherlands; 3 Department of Radiology and Nuclear Medicine, Amsterdam UMC, Amsterdam, The Netherlands; 4 Cardiovascular Biomechanics, Eindhoven University of Technology, Eindhoven, The Netherlands; 5 Computational Science Lab, Faculty of Science, Institute for Informatics, University of Amsterdam, Amsterdam, the Netherlands; Nanjing University, CHINA

## Abstract

Digital twin technologies (DTTs) are increasingly applied in cardiovascular medicine to support personalized treatment. At the same time, growing evidence demonstrates sex differences in cardiovascular anatomy, physiology, disease presentation, and outcomes. Whether current cardiovascular DTTs adequately incorporate these sex-specific characteristics is unclear. This narrative review examines how sex bias and inclusivity are addressed within cardiovascular DTTs and identifies where sex-related bias may arise within different components of DTT. A six-dimensional digital twin framework is introduced to review DTTs. We focus on three cardiovascular domains: coronary artery disease, aortic valve stenosis, and atrial fibrillation. For each domain, we assessed sex representation in modeling assumptions, data interpretation, clinical outputs, and validation studies. Within three domains, physiological assumptions, boundary conditions, interpretation thresholds, and validation cohorts, models are frequently derived from sex-skewed populations. DTTs estimating noninvasive fractional flow reserve are predominantly validated in male-dominated cohorts, potentially resulting in lower precision in women. In contrast, validation studies for DTTs in TAVI planning show variable sex distributions, with some cohorts being female-skewed and others male-skewed, raising questions about their generalizability across sexes. In both model-based DTTs, sex bias may arise from generalized boundary conditions. Electro-anatomical mapping systems for atrial fibrillation are susceptible to sex bias within measurement methodology. Uniform clinical thresholding and outcome selection bias further add to sex-bias in DTTs. Current cardiovascular DTTs insufficiently account for sex-specific cardiovascular characteristics, risking underperformance in underrepresented populations. Incorporating sex-aware physiological parameters, sex-stratified validation, balanced datasets, and transparent reporting should be considered minimal standards for future clinical implementation.

## Author summary

Digital twin technologies (DTTs) are virtual models of patients or organs that help personalize cardiovascular treatments, such as diagnosing heart disease or planning valve replacements. However, these technologies often overlook sex-specific differences in heart anatomy, physiology, and disease presentation, despite growing evidence that men and women experience cardiovascular conditions differently. This narrative review introduces a six dimensional framework and explores how sex bias can enter DTTs at every dimension, from data collection to clinical decision-making. Focusing on clinical available DTTs for coronary artery disease, aortic valve stenosis, and atrial fibrillation, we identify opportunities to improve inclusivity. We propose actionable recommendations, including sex-stratified validation, incorporating sex-specific physiological parameters, and documenting limitations transparently to ensure equal cardiovascular care for all patients.

## 1 Introduction

In healthcare, Digital Twin Technology (DTT) combines computer modeling and clinical data to create a virtual replica of patients, organ systems, or specific physiological subsystems. It is a rapidly growing approach and its potential to improve diagnosis and personalized treatment planning has been especially shown in cardiovascular medicine [1]. At the same time, sex-specific cardiovascular research has gained increasing attention [2] as evidence shows that cardiovascular anatomy [3], physiology [4], and disease presentation [5] as well as cardiovascular disease risk factors [6] differ between men and women. Yet, despite the promise of DTT to deliver personalized healthcare, it is unclear whether current DTT implementations adequately incorporate sex-specific characteristics. Here, we review literature on sex-specific representation within current DTTs.

### 1.1 Sex differences in cardiology

It is increasingly recognized that men and women differ in cardiovascular anatomy, physiology, and disease presentation beyond simple scaling. The heart, for instance, differs in morphology and material properties (e.g., myocardial stiffness and contractile force), which cannot fully be explained by allometric scaling based on (lean) body weight and height [3]. Differences in cardiac morphology and material properties (e.g., weight, cavity and wall volume, and stiffness) result in sex-specific cardiac function (e.g., cardiac output, stress, strain, pressure, heart rate) even before accounting for hormonal and neuroregulatory influences. Indeed, cardiovascular pathology presents differently in men and women, and also prognosis of cardiovascular conditions is different.

During recent years, the clinical impact of sex-specific differences within heart disease has been demonstrated. For example, ischemic heart disease without obstructive coronary artery disease is more prevalent in females (54% female sex), while obstructed coronary artery disease is more prevalent in men (79% male sex) [7]. Furthermore, the female sex seems to be associated with a higher risk of atrial fibrillation-related stroke. But this association could also be affected by the undertreatment of women with oral anticoagulants [8]. In addition, female-specific or -predominate conditions are associated with long-term risk of cardiovascular disease, reflecting differences in underlying mechanisms, such as hypertensive disorders of pregnancy, onset of menarche and menopause, and migraines [6]. Taken together, these examples, ranging from ischemic heart disease and arrhythmias risk to pregnancy-related disorders, demonstrate that sex differences play a significant role in all domains of cardiology. However, only 48% of the ESC guidelines (2018–2023) has a section dedicated to sex-specific medicine and only 1.5% of all references cited within these guidelines are articles explicitly addressing sex differences [9]. Hence, clinical guidelines mainly refer to general physiology without specifically addressing sex differences.

Meanwhile, DTT in healthcare is rapidly increasing and is considered a promising tool for personalized care, for instance by predicting patient-specific treatment outcomes based on medical images. In cardiovascular medicine, where women are still underrepresented in research and clinical trials, accounting for approximately 41% of participants of the 1079 registered cardiovascular trials from 2017 to 2023 [10]. DTTs could either help close or unintentionally increase the gap in diagnosis and treatment between men and women. Note that both biological sex and sociocultural gender affect cardiovascular health [11], but this review solely addresses biological sex.

### 1.2 Digital twin technology

**Fig 1:**
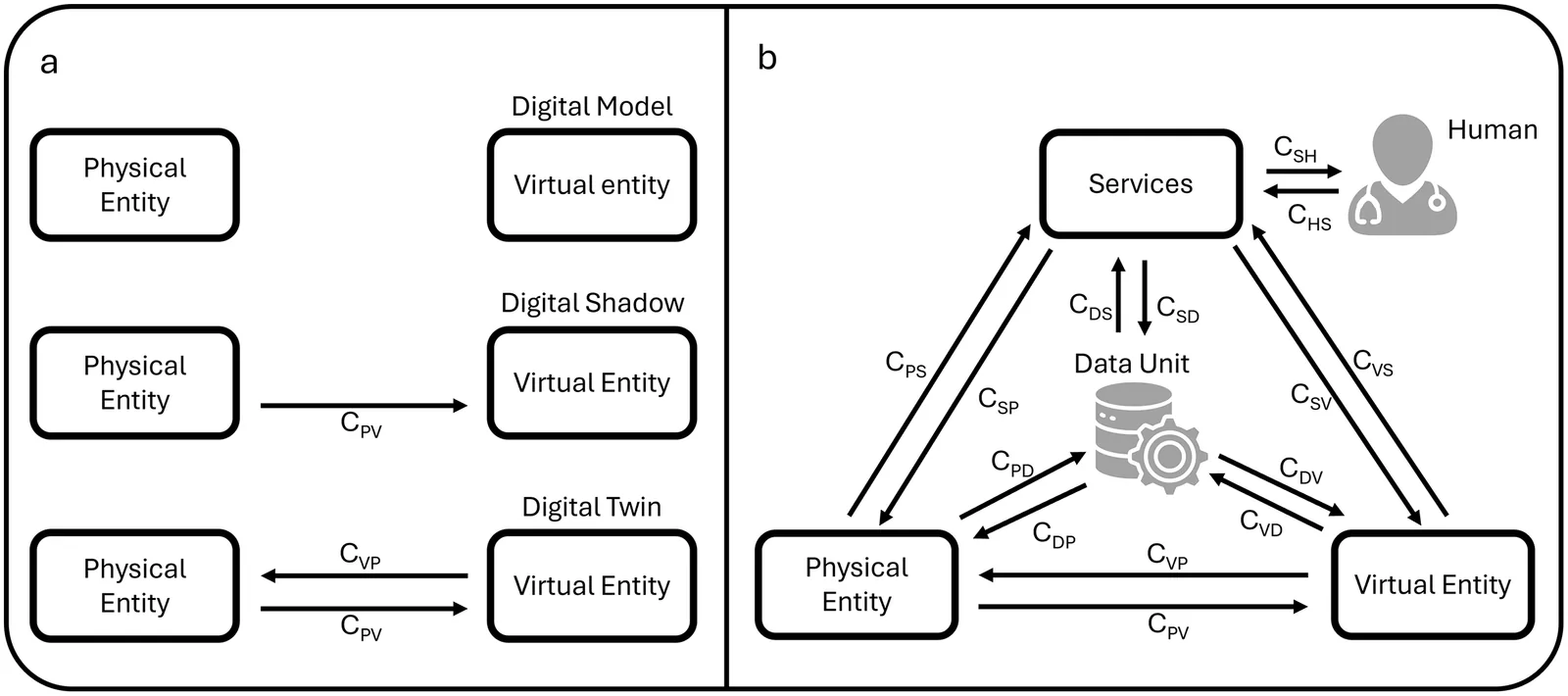
Conceptual classification and overview of Digitial Twin Technologies. a) Schematic representation of the Digital Model, Digital Shadow, and Digital Twin. b) Schematic overview of the proposed 6D framework of a digital twin within healthcare, with *C*_#_ representing connections between five dimensions. This figure is based on Herman et al. (2015) and Tao et al. (2018) [15,16]. The illustration is designed and drawn by the authors. The icons representing the Human and Data Unit are from Flaticon.

VanDerHorn and Mahadevan [12] define a digital twin as *“a virtual representation of a physical system (and its associated environment and processes) that is updated through the exchange of information between the physical and virtual systems.”* While the term ‘digital twin’ is widely used, many reported applications represent only parts of a complete digital twin. To clarify these levels of maturity, Thelen et al. (2022) describe three levels of DTT representation: 1) the Digital Model, a static, isolated virtual representation without automatic data exchange, 2) the Digital Shadow, a model automatically updated from the physical entity but without feedback to it, and 3) the Digital Twin, a model with bidirectional, real-time interaction between the physical and virtual entities [13] (Fig 1a). More recent initiative, the Virtual Human concept proposed within the EDITH project, extends beyond digital twins of a single system and is defined as a “*methodological and technological framework that, once established, will enable collaborative investigation of the human body as a single complex system*” [14]. The complete Virtual Human ecosystem is out of scope for this review.

To develop a true Digital Twin, Tao et al. (2018) proposed a five-dimensional (5D) architecture, demonstrated for the purpose of simulating a wind turbine [15]. This architecture is adapted to healthcare context as illustrated by Herman et al.[16]. In a medical setting, this architecture (Fig 1b) consists of:

1. Physical Entity: the patient, or a specific organ or physiological system.
2. Virtual Entity (Digital Model): a computational model of the physical entity, often built from mechanistic, physics-based descriptions of anatomy and physiology.
3. Services: interfaces and tools (e.g., monitoring systems, clinical dashboards, or decision-support systems) that translate outputs of the Virtual Entity and measurement data of the Physical Entity into clinically actionable information. By visualizing data, this dimension allows for interaction with the digital twin and supports the Human in the Loop in making informed decisions.
4. Data Unit: the integration of patient data (e.g., imaging, physiological measurements, historical records, demographics) with population-based knowledge and data-driven models (e.g., statistical inference, machine learning) to interpret measurements, estimate unobservable variables, and predict outcomes, which can either be shown directly via services or used as input for the virtual representation.
5. Connections: data and information flows linking the other four dimensions, ensuring real-time synchronization, monitoring, and model updating.

To align with the digital twin definition of National Academies of Sciences, Engineering and Medicine [17], we propose a sixth dimension:

6. The Human in the Loop: to emphasize that clinical decision-making remains mediated by human expertise, ensuring ethical oversight.

This review examines potential sources of sex bias in cardiovascular DTTs, which stem from disproportionate weighting of one sex. Two types of bias can be distinguished: 1) prevalence-driven bias (e.g., reflecting natural disease distribution in study population and distribution in patients willing to be enrolled in studies) and 2) systematic bias (resulting from sex-generic design choices, such as uniform physiological models). It further discusses sex inclusivity, referring to the inclusion of individuals of both sexes and accommodation of sex-specific characteristics in the design and application of DTTs for cardiovascular disease. Using the proposed 6D framework allows us to systematically identify where sex bias or underrepresentation can enter a digital twin. DTT can lose inclusivity at every dimension beyond the Physical Entity, which is simply the patient. Once the patient is abstracted into digital data and models, assumptions, simplifications, or imbalances in underlying datasets, such as sex-based underrepresentation, may introduce sex bias. In the Virtual Entity, mechanistic cardiovascular models are often based on population-averaged parameters or on simplified assumptions that physiological differences between sexes can be captured by allometric scaling, despite evidence of the contrary [3]. These assumptions are largely driven by sex-skewed datasets and clinical trial populations used for model development and validation. In the Services layer, visualization of data can be misleading, for instance when clinical thresholds are used for both sexes while the implication of this value depends on sex. Although sex bias in clinical thresholds exists in routine care, in DTTs this bias can be further propagated through feedback loops. Outcomes generated using these thresholds are then used to refine or validate the Virtual Entity, leading models to align with the threshold-based results rather than underlying sex-specific physiology. In the Data Unit, machine learning models use both population-based and patient-specific datasets to give information to services or virtual entity. These population-based datasets are often sex-skewed [18]. While AI models offer approaches to partially mitigate data imbalance, such as re-sampling or cost-sensitive learning, these methods introduce new limitations, including synthetic noise, boundary distortion, and increased model complexity, and do therefore not fully resolve bias [19]. This may lead to less accurate classification or even misclassification of underrepresented groups. Even the Human in the Loop is not bias-free; for instance, women receive catheter ablation therapy less often than men at comparable levels of arrhythmia burden [20]. Finally, once underrepresentation of a sex enters any of these dimensions, the connections that synchronize data and models will propagate, resulting in sex-bias across the digital twin, and feedback loops can further amplify these biases over time.

Many cardiovascular DTTs are, from a physical and mathematical perspective, intrinsically generic between men and women, as the governing equations are sex-independent and, in principle, allow generalization across populations. In practice, however, their performance may become constrained by data availability rather than model structure. Sex-related differences in parameters and boundary conditions are often insufficiently measured or unavailable, which can necessitate simplified or population-averaged inputs. Consequently, even technically sound models may inadequately represent sex-specific behavior when patient-specific measurements are lacking. While this limitation is particularly relevant in physics-based models due to their dependence on parameterization, data-driven AI models face challenges when sex-related differences are underrepresented in training data.

In short, when sex imbalance is present in cardiovascular research and sex-specific physiology is oversimplified or omitted in mathematical models, the accuracy of patient-specific predictions may be compromised, even when patient-specific data are used, risking widening the gap in effective care between women and men.

### 1.3 Aim and scope

This narrative review explores how current cardiovascular DTTs incorporate sex-specific characteristics. To address this aim, we examine three DTT applications to illustrate where sex bias may occur and how inclusivity can be improved.

## 2 Digital twin technologies in cardiology

To assess whether DTTs consider sex-specific characteristics, we exclusively focused on the inclusion of sex-specific characteristics in clinically implemented DTTs with regulatory clearance (FDA clearance or CE marking). These DTTs represent the most mature applications for assessing sex-specific assumptions in cardiovascular care. We examined DTTs developed for three cardiovascular diseases: coronary artery disease, aortic valve stenosis, and atrial fibrillation, that have been widely explored in DTT research [21], each relying on distinct mechanistic modeling approaches. In this narrative review, we explore potential sex-related bias arising from design and validation choices in these DTTs.

### 2.1 Coronary artery disease

Coronary artery disease is the leading cause of death [22], with reopening of the vessel with a stent or bypassing the stenosis with a coronary artery bypass graft as the most common treatments [23]. Choosing which treatment as well as which coronary vessel and where in the vessel to treat can be challenging because this disease is often diffuse, lesions can be tandem or long, and multiple vessels may be affected, which is even more relevant in (older) women [24]. The clinical index fractional flow reserve (FFR) is used to quantify the functional severity of stenoses. However, as FFR is measured invasively, patients are exposed to procedural risks. To avoid those risks, DTTs for coronary artery disease provide noninvasive FFR surrogates. Several of these DTTs have obtained FDA 510(k) clearance and CE marking (e.g., HeartFlow [25], Medis [26], CathWorks [27], Pie Medical Imaging [28], and Keya Medical [29]).

**Fig 2:**
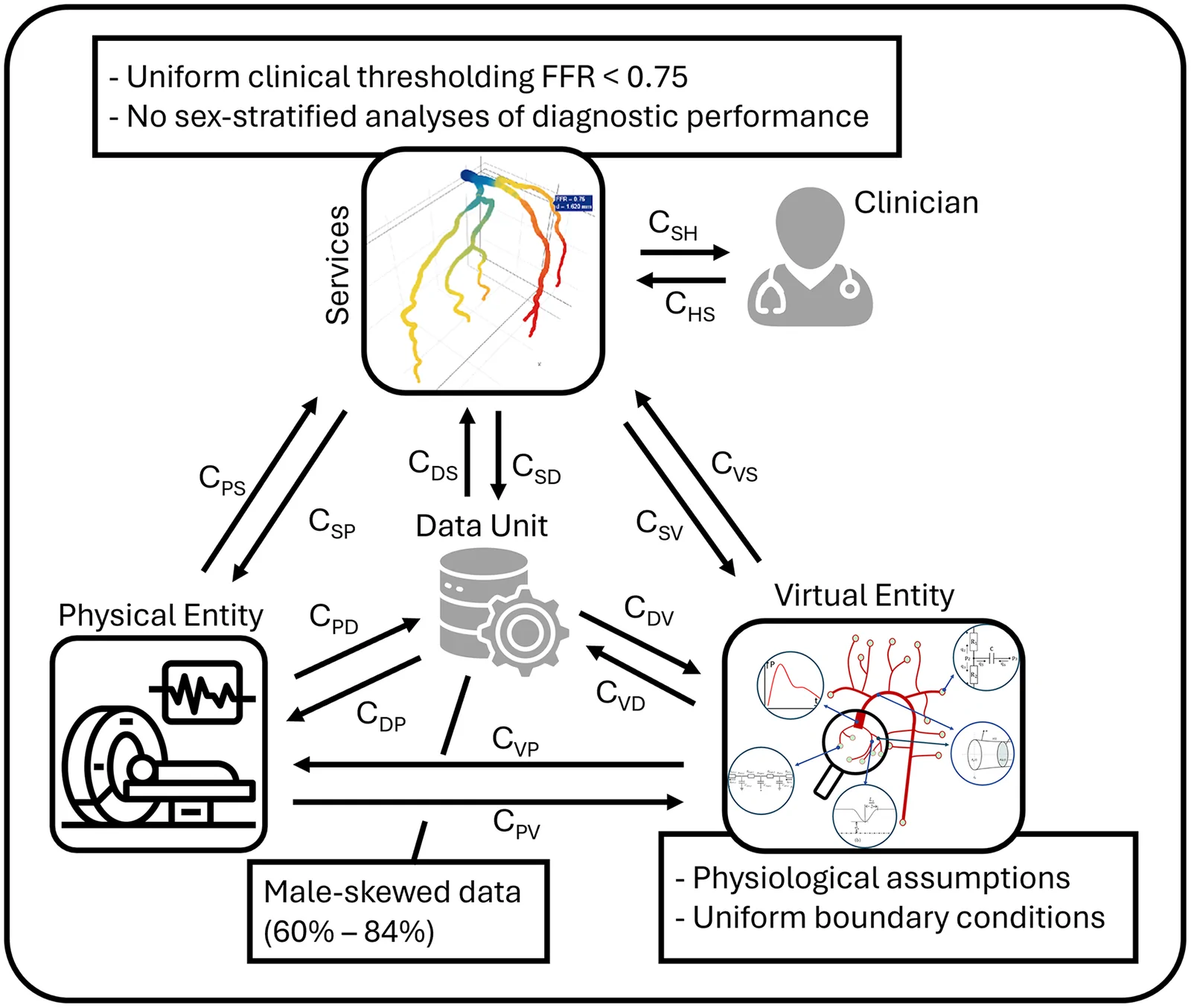
Schematic overview of data and information flows in DTTs estimating the FFR. The figure illustrates how data are transferred between Physical Entity, Virtual Entity, Data Unit, and Services. Table 1 gives an explanation of all connections. Boxes highlight potential sources of sex-related bias, including data-driven limitations (e.g., male-skewed input data), model-related assumptions (e.g., physiological parameters or uniform boundary conditions), and clinical decision-making (e.g., uniform clinical thresholding). The illustration is designed and drawn by the authors. The icons representing the Human, Data Unit and Physical Entity are from Flaticon.

Although sex bias in clinical (Fig 2), the Physical Entity is the patient’s coronary anatomy, imaged via coronary computed tomography angiography (CCTA) [29,30] or coronary angiography [26,27,31]. The Virtual Entity is a patient-specific digital representation of the coronary arteries, in which coronary geometry is used to estimate blood pressure and flow, and thus derive FFR [28,31–33]. To generate a Virtual Entity, the coronary arteries are segmented from CCTA or one or multiple 2D coronary angiography images using semi-automatic or machine learning-assisted workflows as part of the Data Unit. The resulting geometry is used, by the Virtual Entity, to estimate pressure and flow in the coronary tree using computational fluid dynamics (CFD) (e.g., HeartFlow), reduced-order models describing relations between anatomy, flow, and pressure (e.g., Medis, Pie Medical, CathWorks), or Deep Learning algorithms informed by CFD (e.g., Keya Medical). These pressure and flow estimations are used to compute the FFR. Besides the segmentation, the Data Unit provides and combines patient-specific imaging data, patient characteristics, and population-based physiological parameters (e.g., boundary conditions) to parameterize the Virtual Entity. Services visualize the resulting FFR values to support clinical decision-making, typically using color-coded (i.e., red for values below the threshold; FFR < 0.75). The Human in the Loop, e.g., an interventional cardiologist, can use this information to make a treatment plan. The Connections between these dimensions (Table 1) facilitate information exchange, including patient demographics (C_PS_, C_PV_), imaging data (C_PD_), and model computations (C_DV_, C_VS_), as well as comparing model outcomes with measurements from Physical Entity to refine or validate the model (C_VP_, C_DP_). The extracted geometry is patient-specific and captures sex-specific anatomical differences, including vessel size and myocardial mass. However, while boundary conditions and physiologic parameters could be patient-specific, they are often population-averaged in current implementations and typically validated using male-dominated reference data [28,31–33]. This particularly relevant given that for the same degree of angiographic stenosis, women show higher measured FFR values compared to men, reflecting lower coronary blood flow, differences in microvascular function, and myocardial mass [34]. Because clinical decision-making relies on a fixed FFR threshold (FFR < 0.75 [35]) to indicate ischemia, these physiological differences, together with population averaged parameters, may lead to fewer women crossing the diagnostic cutoff. This may inturn contribute to their underdiagnosis, particularly in women with diffuse or multivessel disease, where lesion-specific FFR may not fully capture the combined hemodynamic burden [34].

**Table 1:** Interpretation of the connections within the 6D-architecture for DTTs for noninvasive FFR, TAVI procedure, and ablation guidance for AF.

		FFR	TAVI	AF
Connections originating from Physical Entity	**C**_**PV**_	Patient demographics		
	**C**_**PD**_	Coronary imaging data	CT imaging	EAMs
	**C**_**PS**_	Patient demographics		
Connections originating from Virtual Entity	**C**_**VP**_	Model calibration		
	**C**_**VD**_	Save FFR computations	Save contact pressures, coronary obstruction and/or valvular leakage	Save locations and electrograms
	**C**_**VS**_	Computed FFR per location	Simulated contact pressure, paravalvular leakage, or coronary obstruction.	Electrical conduction maps
Connections originating from Services	**C**_**SP**_	Explanation to patient		
	**C**_**SD**_	Save interaction with clinician	Save interaction with clinician	Save interaction with clinician
	**C**_**SV**_	Treatment decision	Input of valve type, size, and implementation depth	Ablation sides
	**C**_**SH**_	Visualization of FFR through the coronary arteries	Visualization of contact pressure, paravalvular leakage, and coronary obstruction.	Visualization of 3D mesh as well as voltage, activation, and propagation map
Connections originating from Data Unit	**C**_**DP**_	Data calibration		
	**C**_**DV**_	Segmented coronary arteries and myocard	Segmented aortic root and valve leaflets	3D interpretation of atria, catheter positions, and voltage interpolation
	**C**_**DS**_	Segmented coronary arteries	Segmented aortic root and valve leaflets	3D interpretation of atria and catheter positions
Connections originating from Human in the loop.	**C**_**HS**_	Treatment decision	Virtual tests of valve type, size, and implementation depth.	Decision in ablation side.

The DTTs that use simplified relations between anatomy, flow, and pressure (Medis, Pie Medical Imaging, and CathWorks) estimate pressure drop across coronary stenoses based on vessel geometry and empirical coefficients for viscous friction and flow separation. The original derivation of these coefficients has not been transparently reported, but their validation studies consistently included a male majority, reflecting sex differences in incidence. For the product of Pie Medical Imaging, the initial FAST study included 67% men [31], FAST-Extend 66% [33], and FAST-2 73% [31]. For the product of Medis, a meta-analysis of 16 studies reported male inclusion rates ranging from 60% to 84% [36]. Similarly, CathWorks’ validation is based on predominantly male cohorts, with a meta-analysis of five studies showing 72% men [27].

Similar to the validation of DTTs based on simplified relations, CFD-based solutions are validated on sex-skewed database as well. A meta-analysis of five HeartFlow validation studies reported male inclusion ranging from 60% to 76% [30]. For validation of the product of Keya Medical, the only peer-reviewed publication to date included a nearly balanced cohort (51% men) of only 63 patients [29]. A CFD solution relies on prescribed boundary and initial conditions, including inlet pressure or flow waveforms, outlet conditions such as fixed pressure, resistive or Windkessel models, no-slip wall assumptions, and initial velocity fields. While these boundary conditions differ between sex, neither company has presented sex-stratified analyses of diagnostic performance. Although the exact definitions of these boundary conditions are not disclosed by the companies, it is likely that allometric scaling has been applied [37]. However, scaling does not capture sex differences in physiology and pathophysiology, such as differences in microvascular resistance [38], myocardial mass [3], vessel compliance [39], regulation mechanisms of blood pressure [40], and disease presentation [41].

### 2.2 Aortic valve stenosis

The prevalence of aortic valve stenosis is expected to continue to increase with the aging population [42,43]. Transcatheter aortic valve implantation (TAVI) and surgical aortic valve replacement (SAVR) are the common treatment options [44]. Choosing the appropriate valve type and size and predicting procedural risks, such as paravalvular leak or conduction abnormalities, are challenging due to heterogeneous anatomy, varying calcification patterns, and proximity of the coronary ostia [45]. To support TAVI planning, DTTs predict simulate TAVI device implantation and predict complications patient-specific.

**Fig 3:**
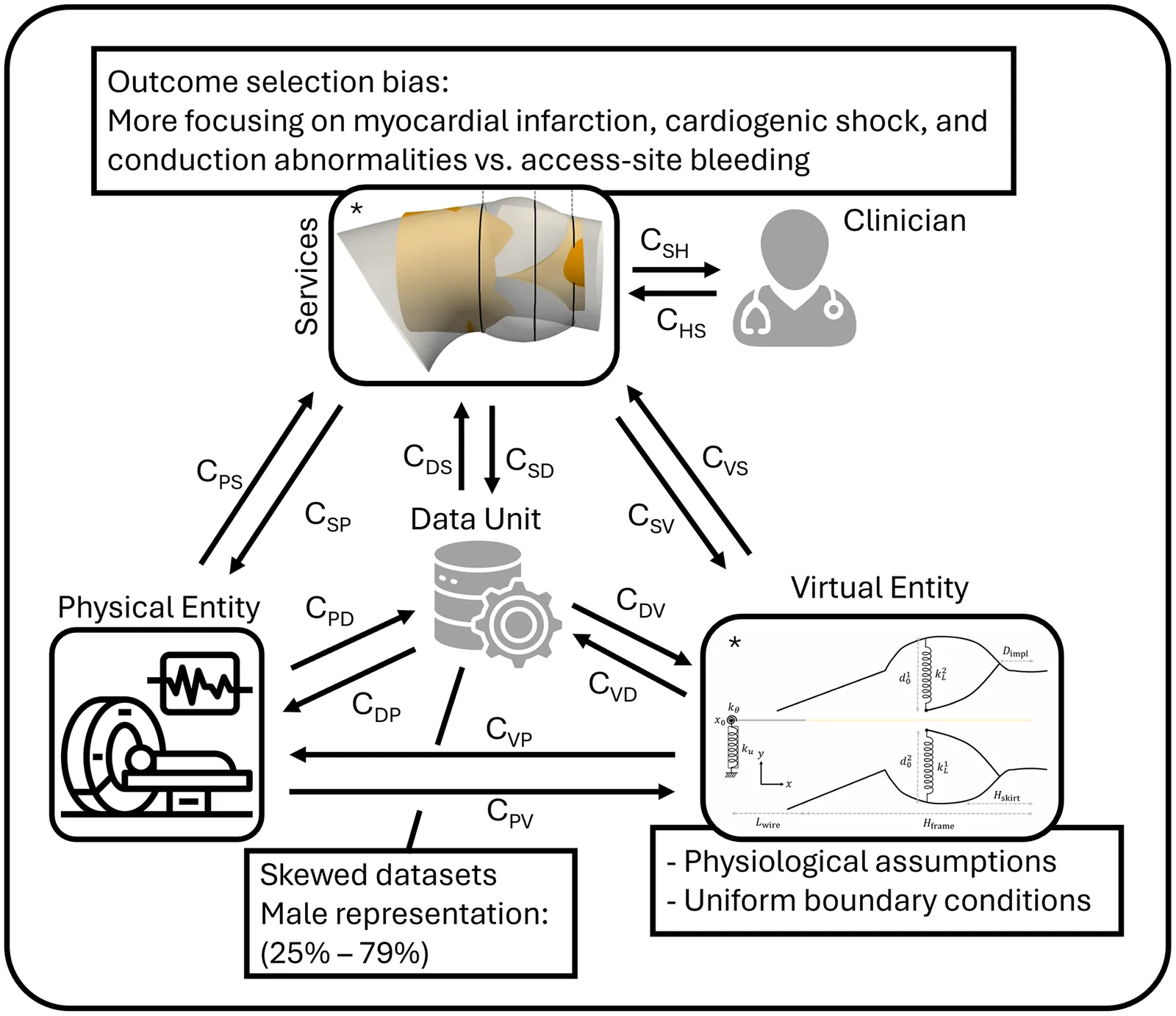
Schematic overview of data and information flows in DTTs for TAVI planning. The figure illustrates how data are transferred between Physical Entity, Virtual Entity, Data Unit, and Services. Boxes highlight potential sources of sex-related bias, including data-driven limitations (e.g., sex-skewed input data), model-related assumptions (e.g., uniform boundary conditions or physiological parameters), outcome selection bias. The illustration is designed and drawn by the authors. The icons representing the Human, Data Unit, and Physical Entity are from Flaticon. *Images are adopted from the PhD thesis of S.C.F.P.M. Verstraeten [63] with permission.

In such DTTs for TAVI planning (Fig 3), the Physical Entity is the patient’s aortic root and valve. The Virtual Entity is the virtual replica of the patient’s aortic root and valve using modeling techniques to simulate TAVI device deformation during the procedure, predict structural changes of the aortic root such as stress and strain, and estimate intervention procedure complications including damage of aortic root, paravalvular leakage, coronary obstruction, and/or device migration [46,47]. Typically three modeling approaches are used: Finite Element Analysis (FEA), CFD, and Fluid Structure Interaction (FSI). The Data Unit segments the aortic root and valve leaflets (including calcification) based on CT using semi-automatic workflows (e.g., software Mimics of materialize, Materialise Inc., Leuven, Belgium or VMTK). These segmentations are supplied to the Virtual Entity together with assigned tissue thickness and material properties to the aortic wall and valve leaflets using constitutive models such as isotropic neo-Hookean hyper-elastic model [48] or the Mooney–Rivlin hyperelastic constitutive law [49]. Using Services, a clinician can virtually implant a digital stent. The Virtual Entity mimics pre-dilation, valve deployment, and post-dilation. The resulting stress, strain, and deformation of the aortic root due to TAVI are calculated by applying FEA to a patient-specific 3D model of the aortic root and valve, generated from CT-based segmentations. To assess paravalvular leakage, the deformed geometry can be analyzed by simulating blood flow using CFD under applied boundary conditions such as diastolic pressure gradient. The effect of multiple valve types, sizes, and implantation depths can be explored to predict for instance contact pressures [50], coronary obstruction risk [51,52], and valvular leakage [50]. These simulated outcomes are subsequently visualized to support clinical decision making by Services. The Connections between these dimensions (Table 1) exchange of information. Examples of information flows are valve type, size, and implementation depth values chosen by the clinicians to perform virtual tests (CHS, CSV, CVS). The Human in the Loop makes the final decisions on valve type, size, and implantation depth.

Two commercial platforms have emerged: FEops HEARTguide and DASI Simulations (PrecisionTAVI). FEops HEARTguide combines FEA and CFD to support the selection of valve size and estimation of implantation depth based on predicted paravalvular leakage and contact pressures following TAVI procedure. Retrospective validation studies of HEARTguide include cohorts ranging from 9 to 112 included patients. In these studies, male representation varied from 25% to 57% [53–56] and one study did not report sex distribution [57]. El Faquir et al. reported a prospective validation study including 80 patients, with simulations performed in a subset of 42 patients, of whom 79% were male [51]. In a more recent prospective study by Hokken *et al*. simulations were conducted in 77 patients, with an underrepresentation of men (42% male participants) [50]. DASI Simulations (PrecisionTAVI) applies FEA to predict coronary obstruction risk and to support the selection of valve type, size, and implantation depth. PrecisionTAVI was validated in a prospective multicenter study including 116 patients, of whom 90 underwent TAVI, with 36% being male [58]. In addition, a retrospective single-center study by Sirset-Becker et al. performed simulations in a subset of 32 out of 74 patients; however, the sex distribution of this cohort was not reported [52]. Thus, validation cohorts for DTTs for TAVI planning show variable sex distribution from male- to female-skewed.

Similar to the DTTs for estimating noninvasive FFR, patient-specific geometry is incorporated in the model, thereby capturing differences in annulus size, aortic root geometry, calcification patterns between men and women [59]. Simulations could therefore potentially help reducing the mismatch between prosthesis and patient aortic dimensions, for example by accounting for the altered pressure recovery associated with the smaller aortic root often observed in women [59]. However, current digital twin models still assume population-based uniform aortic root thickness [47], uniform or algorithmically assigned leaflet thickness [47], and uniform tissue stiffness. Although patient-specific geometry is incorporated, material properties such as aortic wall thickness, leaflet thickness, and tissue stiffness are typically prescribed using uniform or population-based values. As a result, inter-individual and sex-specific variations in aortic wall mechanics and pressure recovery are not captured [59].

In addition, outcomes after TAVI differ between sexes. Women more often experience in-hospital mortality, stroke, bleeding, and vascular complications, whereas men more frequently develop cardiogenic shock and acute kidney injury [60,61]. Current DTTs for TAVI planning focus on contact pressures [50], coronary obstruction risk [51,52], and valvular leakage [50], thereby focusing on outcomes such as myocardial infarction, cardiogenic shock, and conduction abnormalities. However, access-site bleeding remains a common complication after TAVI and is more often major or life-threatening in women than in men (51% vs. 23%; *p* < 0.01) [62]. Consequently, complications that disproportionately affect women, such as bleeding and vascular injury, are less frequently represented among modeled outcomes (outcome bias).

### 2.3 Atrial fibrillation

Atrial fibrillation (AF) is the most common sustained arrhythmia with a lifetime risk affecting up to one in three individuals with prevalence increasing with age and comorbidities such as hypertension, obesity, and heart failure [64–66]. AF is associated with a substantially increased risk of stroke, heart failure, myocardial infarction, dementia, and mortality [66]. Rhythm control with catheter ablation is one of the main therapies to restore and maintain normal sinus rhythm. Yet, the choice of ablation target remains challenging due to heterogeneous mechanisms of AF drivers [64]. Electro-anatomical mapping systems (EAMs) guide ablation procedures by visualizing measured electrical activation patterns. In contrast to model-based DTTs used for TAVI planning or FFR estimation, EAMs translate measured electrograms into clinically actionable maps without incorporating a physiological simulation model. The three most commonly used EAMs are Carto 3 (Biosense-Webster, Johnson and Johnson, Inc.), Ensite Precision (Abbott, Inc.), and the Rhythmia Mapping system (Boston Scientific, Inc., Marlborough, MA) [67].

**Fig 4:**
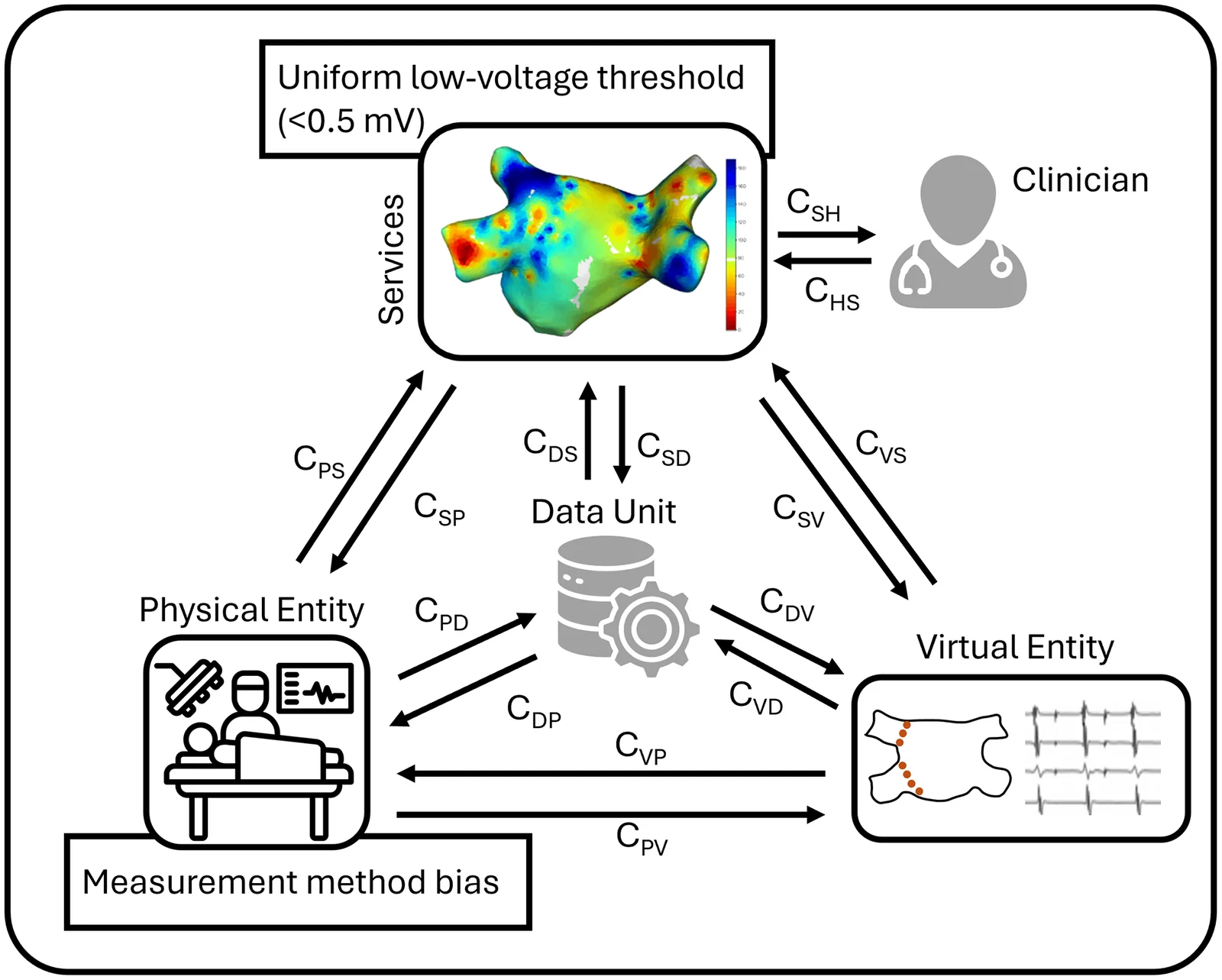
Schematic overview of data and information flows in EAMs. The figure illustrates how data are transferred between Physical Entity, Virtual Entity, Data Unit, and Services. Boxes highlight potential sources of sex-related bias, including data-driven limitations (e.g., measurement method bias) and clinical decision-making (e.g., uniform clinical thresholding). The illustration is designed and drawn by the authors. The icons representing the Human, Data Unit, and Physical Entity are from Flaticon.

In EAMs to guide ablation (Fig 4), the Physical Entity is the patient’s atrial anatomy and electrical activity. The Virtual Entity is a digital replication of the atrial anatomy and electrical activity. The Data Unit reconstructs patient-specific atrial anatomy by localizing catheter positions in three-dimensional space and provides the Virtual Entity this generated atrial anatomy with the measured unipolar or bipolar electrograms, including their locations within the atrial anatomy [68]. The Virtual Entity generates left atrium voltage-, activation-, and propagation- maps from the resulting electrogram signals, spatial coordinates of the signals, and reconstructed geometry. Voltage maps can be used as a surrogate to identify fibrotic tissue. Activation maps can be used to track wavefront propagation, propagation maps display temporal activation. Services visualize voltage maps. The Human in the Loop, typically a cardiologist, interprets the maps to plan ablation treatment as a reduced bipolar amplitude is assumed to reflect fibrotic substrate and activation and propagation maps can be used to visualize re-entry circuits. The Connections between these elements (Table 1) ensure the exchange of information, such as catheter location and local electrograms (C_DV_, C_SH_, C_DS_).

The resulting EAMs depend on acquisition conditions, such as the reference location and timing window, catheter–tissue contact, spatial sampling density, and atrial geometry, as well as on interpretation assumptions, including the definition of low-voltage thresholds. Bipolar voltage, for example, are influenced by wall thickness, atrial size, catheter orientation, activation direction, and electrode contact [69]. Despite this complexity, in clinical practice, a uniform low-voltage threshold (<0.5 mV) is commonly used to identify fibrotic substrate and, thus, potential AF driver zones. However, multiple studies have shown that atrial voltages are significantly lower in women than in men and that women exhibit more low-voltage zones [70–72], reflecting sex-related differences in atrial size, wall thickness, and histopathology [73]. As a result, the use of uniform voltage thresholds may systemically overestimate fibrotic substrate in females.

Taken together, these findings suggest that sex-related biases in EAMs are more likely introduced through measurement methods, such catheter size, and clinical thresholding rather than boundary conditions and validation studies as seen in model-based DTTs. This sex-based difference in atrial voltage suggests that current voltage thresholds may systematically overestimate fibrotic substrate in female patients.

## 3 Discussion

DTTs and sex-specific cardiovascular research are both rapidly growing; however, this review demonstrates that the integration of these fields is currently insufficient. The noninvasive FFR and AF driver indication software are largely validated on male-skewed data, reflecting disease prevalence in men and women (prevalence-driven bias). In addition, these DTTs are built upon male-representative physiological models (systematic bias). In contrast, validation studies for TAVI simulation software show variable sex distributions, with some cohorts being female-skewed (e.g., 75% female in [55]) and other male-skewed (e.g., 79% male in [51]). This is relevant because of different sex specific anatomical and physiological features between men and women, and because of different treatment outcomes. All DTTs rarely account for sex-specific physiological, tissue, or anatomical characteristics as well as measurement methods. Within the domains of the three cardiovascular diseases, coronary artery disease, aortic valve stenosis, and atrial fibrillation, we discuss opportunities to enhance inclusivity in current DTT.

### 3.1 Opportunities

#### 3.1.1 Coronary artery disease.

The underlying male-skewed validation and most probable male-based model representation of the DTTs that estimate FFR result in a lower precision of performance for the female population. Under the simplifying assumption of comparable variability between sexes, the statistical precision of estimated performance metrics scales with the inverse square root of the sample size. For a representative 70:30 male-female ratio, this results in approximately 50% lower precision in performance estimates for women compared to men. To date, however, no study has explicitly documented differences in classification performance of noninvasive FFR between men and women.

While male predominance in current validation studies reflects the higher prevalence of obstructive CAD in men [74], it does not justify the lack of sex-stratified validation. To ensure that models perform equally well in women, deliberate inclusion strategies (e.g., oversampling of women) are required regardless of prevalence. Moreover, women with obstructive CAD are already at risk of underdiagnosis: they more often present with atypical symptoms [75], show higher FFR values for the same degree of angiographic stenosis [34], and undergo PCI less frequently than men with comparable disease burden [76]. Hence, this sex bias within the DTTs that estimate FFR in healthcare may reinforce this cycle in which female patients are less likely to be recognized, less likely to be included in validation studies, and less likely to receive treatments.

#### 3.1.2 Arotic valve stenosis.

In contrast to DTTs that estimate FFR, DTTs for TAVI planning are often validated on cohorts with a higher proportion of female patients, reflecting the demographic distribution of the TAVI population. As a result, performance estimates are less precise for male patients due to their underrepresentation in validation datasets. This reduced precision, arising from sex bias in cohort composition, highlighting the need to assess whether current digital twin approaches for TAVI planning adequately capture outcome differences between men and women.

As outlined in Section 2, clinical outcomes after TAVI differ substantially between women and men, while current DTTs primarily target a limited subset of procedural and device-related endpoints. This mismatch indicates that current DTTs for TAVI planning may not capture the outcomes that are most relevant for both sexes (outcome selection bias).

Typically, the valve prosthesis is delivered via a catheter inserted through the femoral artery, advancing along the iliac arteries and the aorta towards the aortic valve. Current DTTs focus on the aortic root, valve leaflet dynamics, and local hemodynamics around the prosthetic valve, while ignoring this distal vascular trajectory. Because access-site vascular complications remain a major cause of TAVI-related morbidity, incorporating imaging of this pathway and CT-based vessel distensibility to capture vessel morphology and stiffness could improve prediction of perforation risk and enable patient-specific procedural strategies, including access-site planning, and ultimately reduce life-threatening bleeding. Anatomical factors such as smaller vessel diameter, thinner vessel wall, and more tortuous vessels in women, likely contribute to a higher risk of vascular injury, perforation and subsequent bleeding complications [77].

Incorporating sex-specific wall thickness, vessel stiffness, and complete vascular trajectory into DTTs may potentially not only improve prediction of mismatch of valve and patient aortic dimensions but also help anticipate bleeding risk and guide safer device design and deployment strategies for both sexes.

#### 3.1.3 Atrial fibrillation.

The basic mechanisms underlying AF are re-entry and triggered activity, both of which are promoted by atrial fibrosis. Two main types can be distinguished: replacement fibrosis, where dead myocytes are replaced by scar tissue, and reactive fibrosis, where myocytes remain intact but excess collagen is deposited between them (interstitial and endomysial fibrosis) [78,79]. While replacement fibrosis forms discrete scars and conduction blocks, reactive fibrosis leads to subtle conduction slowing and complex atrial electrograms. Importantly, recent studies indicate that endomysial fibrosis, rather than the total fibrotic burden, is primarily responsible for conduction abnormalities [78].

Mapping techniques, however, are unable to capture endomysial fibrosis. Low-voltage areas provide only indirect markers of fibrosis and show inconsistent correlation with histological findings [80]. Because endomysial fibrosis is more prevalent in women, pathophysiological changes may go unnoticed more often and these limitations may lead to sex-related differences in driver identification and ablation outcomes [81].

Sex differences in AF include epidemiology, pathophysiology, and outcomes [82]. Women have a lower lifetime risk of experiencing AF but a higher overall prevalence due to longer life expectancy. Women experience greater symptom burden and face higher risks of stroke, heart failure, and mortality. They are referred later for rhythm-control therapy, less often undergo ablation, and when treated, have higher recurrence rate [83] and slightly increased procedural risk [84], partly explained by smaller vasculature, smaller atria, thinner walls, more diffuse fibrosis, and nonpulmonary vein triggers.

To improve inclusivity, DTTs could integrate multimodal data such as voltage, electrogram fractionation, and wall thickness, incorporating parameters that reflect endomysial fibrosis to better identify AF drivers that are more prominent in women. In addition, integrating energy-delivery simulations that account for patient-specific atrial wall thickness could further optimize ablation safety. Together, such integration would improve the accuracy and inclusivity of AF ablation planning.

### 3.2 Towards sex-aware digital twin technologies

To move beyond generic calls for balanced datasets, we propose actionable recommendations to address sex-related bias in cardiovascular DTTs. These recommendations are structured according to the 6D framework.

#### 3.2.1 Physical entity.

Clinicians, developers, and researchers should ensure that patient measurements used to create a DTT are representative for both sexes. Literature should be reviewed to identify anatomical and physiological parameters relevant to the clinical case of interest, including known sex differences. Measurement methods (e.g., medical imaging, vital signal measurements, body examinations) should then be evaluated to determine whether these parameters capture anatomical and physiological parameters for both sexes, including quantification of measurement uncertainty (e.g., due to imaging resolution or signal noise). The measured parameters should be verified to fall within sex-specific physiological ranges reported in the literature. If these parameters fall outside the physiological range, measurements should be redone or measurement protocols should be adapted or flagged. Any limitations in sex-stratified measurement protocols should be addressed, and limitations, including sex-specific uncertainty, should be transparently documented.

#### 3.2.2 Virtual entity.

To avoid systematic bias, the Virtual Entity should account for sex-specific physiology. Uniform parameters (e.g., tissue properties, hemodynamic boundary conditions) may not capture sex differences, leading to inaccurate simulations. Sensitivity analysis should be performed to identify the most influential parameters and boundary conditions on model outputs and to assess whether their effects differ between sexes. The results of this analysis can guide which parameters require precise measurement whenever feasible and for which parameter population-average estimation is sufficient. Parameters for which sensitivity differs significantly between sexes should be modeled using sex-specific values or distributions rather than uniform defaults. Uncertainty should be quantified (e.g., Bayesian inference), particularly when data is sex-skewed. The use of uniform parameter defaults should be transparently documented and justified.

#### 3.2.3 Services.

Services translate model outputs into clinically actionable information (e.g., treatment recommendations, risk stratification) and must avoid bias. Fixed clinical thresholds and uniform visualization methods (e.g., color-coded maps) may lead to misinterpretation model outputs and propagate bias through feedback loops. These feedback loops should be used to assess and refine clinical thresholds that are validated against sex-stratified clinical outcomes. Even when continuous outputs are presented, design choices such as color scales, alerts, or categorizations should be evaluated for their influence on interpretation. Uncertainty in outputs should be explicitly visualized, and the Human in the Loop should be alerted to underlying assumptions.

#### 3.2.4 Data unit.

Biases within the Data Unit can arise from sex-skewed datasets, inappropriate uniform assumptions about physiological parameters, or population-based values for anatomical or functional characteristics that do not account for sex differences. Sex-balanced datasets should be used for refining and validation. If balanced data is unavailable, sex-stratification methods, such as algorithmic weighting, undersampling majority groups, oversampling minority groups (e.g., using SMOTE), and stratified cross-validation should be applied. Sensitivity analysis can further help identify which input parameters most influence model outputs and, therefore, should be prioritized for accurate measurement. Subsequently, in silico trials should test model performance for various virtual populations with diverse sex-specific characteristics. Additionally, uncertainty in data inputs (e.g., measurement variability, missing data) must be quantified and limitations of the dataset, such as underrepresentation in population data or the use of generic defaults, and the mitigation strategy employed should be transparently documented.

#### 3.2.5 Connections.

To prevent propagation of bias through the Connections, the impact of a feedback loop should be tested on model outputs separately for men and women using sex-stratified datasets. Bias amplification should be quantified by comparing model updates across iterative cycles; divergence in model behavior or performance between sexes should trigger revision of model parameters, thresholds, or data inputs.

#### 3.2.6 Human in the Loop.

The Human in the Loop must be aware of sex-specific limitations in DTT outputs. Interfaces should explicitly present sex-specific uncertainties and assumptions (e.g., through confidence intervals or visual flags) to support informed clinical decision-making. Clear documentation of model assumptions, parameter choices, and data limitations is essential to ensure transparency and appropriate interpretation. The impact of skewed datasets in DTTs estimating the FFR can be roughly estimated, as validation cohorts are consistently male-skewed (~70% men) [27,29–31,33,36], allowing us to estimate a ~50% lower precision for women. In contrast, validation cohorts for DTTs used for TAVI planning show high variability in sex distribution (25%–79% men) [53–56], and DTTs to guide AF ablation lack formal validation studies altogether, as they primarily involve interpreting measurements without a gold standard. As a result, quantifying the overall impact of sex bias in DTTs is not feasible due to this heterogeneity in populations and outcome measures*.*

## 4 Conclusion

Current cardiovascular DTTs are largely calibrated on sex-skewed data and assumptions, risking systematic underperformance in women or men depending on the disease context. Through coronary artery disease, aortic valve stenosis, and atrial fibrillation, this review demonstrates that patient-specific geometry alone is insufficient, and that sex-specific physiological parameterization, clinical decision thresholds, visualization strategies, and sex-stratified validation are required for equitable model performance. These sex-specific modeling elements, together with balanced validation cohorts, should therefore be considered minimal standards for clinical implementation of cardiovascular DTTs.
